# Comparison of Benchtop Fourier-Transform (FT) and Portable Grating Scanning Spectrometers for Determination of Total Soluble Solid Contents in Single Grape Berry (*Vitis vinifera* L.) and Calibration Transfer

**DOI:** 10.3390/s17112693

**Published:** 2017-11-22

**Authors:** Hui Xiao, Ke Sun, Ye Sun, Kangli Wei, Kang Tu, Leiqing Pan

**Affiliations:** College of Food Science and Technology, Nanjing Agriculture University, Nanjing 21009, China; hui.x.1010@outlook.com (H.X.); sk61026@126.com (K.S.); 2015208018@njau.edu.cn (Y.S.); 2016108043@njau.edu.cn (K.W.); kangtu@njau.edu.cn (K.T.)

**Keywords:** near-infrared spectroscopy, total soluble solid contents, calibration transfer, standardization samples selection, linear interpolation-piecewise direct standardization

## Abstract

Near-infrared (NIR) spectroscopy was applied for the determination of total soluble solid contents (SSC) of single Ruby Seedless grape berries using both benchtop Fourier transform (VECTOR 22/N) and portable grating scanning (SupNIR-1500) spectrometers in this study. The results showed that the best SSC prediction was obtained by VECTOR 22/N in the range of 12,000 to 4000 cm^−1^ (833–2500 nm) for Ruby Seedless with determination coefficient of prediction (R_p_^2^) of 0.918, root mean squares error of prediction (RMSEP) of 0.758% based on least squares support vector machine (LS-SVM). Calibration transfer was conducted on the same spectral range of two instruments (1000–1800 nm) based on the LS-SVM model. By conducting Kennard-Stone (KS) to divide sample sets, selecting the optimal number of standardization samples and applying Passing-Bablok regression to choose the optimal instrument as the master instrument, a modified calibration transfer method between two spectrometers was developed. When 45 samples were selected for the standardization set, the linear interpolation-piecewise direct standardization (linear interpolation-PDS) performed well for calibration transfer with R_p_^2^ of 0.857 and RMSEP of 1.099% in the spectral region of 1000–1800 nm. And it was proved that re-calculating the standardization samples into master model could improve the performance of calibration transfer in this study. This work indicated that NIR could be used as a rapid and non-destructive method for SSC prediction, and provided a feasibility to solve the transfer difficulty between totally different NIR spectrometers.

## 1. Introduction

Grape berry (*Vitis vinifera* L.) is one of the oldest and most widely cultivated plants. In recent decades, the production of grapes has continued to increase globally, making it one of the important species of fruit. There are several reasons behind the increasing popularity of the table grape in the summer season, including its great taste and abundance of nutritional ingredients. Total soluble solids contents (SSC) is one of the main indicators of the internal quality of grape berry and it also has a very important influence on the grape taste. The appropriate harvesting time of grape should be defined according to the SSC. Also, due to the different sugar contents of the grapes, different brewing processes have to be performed accordingly. It is necessary to strictly control the soluble solids content of the grape berries during wine-making [1]. 

Near-infrared spectroscopy (NIRS) is a recently popular nondestructive analytical method which does not require any reagents or sample preparation. NIR covers the spectral range from 780 nm to 2500 nm. The vibration behavior of molecules such as stretch-bend combination modes and overtone bands can be found in this spectral range [2]. As reported in the literatures, NIR technology has been successfully implemented for the determination and quantitative analysis of chemical components and physical properties of various samples [3,4,5,6,7,8,9,10]. NIR, by virtue of its speed and non-destructive nature, is advantageous over the traditional method of manual selection performed by experienced winemakers [11].

Chemometric methods are essential tools that can help account for the overlapping of absorption peaks of diverse functional groups. A considerable number of models would be generated at the conclusion of regression. And a key concern in application of NIR and other non-destructive technologies is that the prediction results are influenced by the types of instruments which could cause baseline drift, wavelength drift, and absorbance fluctuation [12], and it makes the model based on one instrument could not be used in other instruments. Thus, it is the key problem when NIR technology comes to industry. To solve this specialized problem, many researchers have raised several calibration transfer methods including slope and bias correction (SBC) [13], Shenk’s Algorithm [14], and piecewise direct standardization (PDS) [15]. The PDS is perhaps the most successful calibration transfer technique and is widely used on commercial portable NIR devices to solve instruments’ drift. However, it is more complex when facing the instruments with diverse types of monochromators and detectors, especially when these instruments have different wavelength resolutions. Several studies have been focused on preprocessing methods and sample selection before transfer to optimize the accuracies of transfer methods, but few research have optimized the method of wavelength correction before transfer. Here, we proposed a simple but practical wavelength correction method before PDS called linear interpolation-PDS by regarding the relationship between two nearest-neighbor wavelengths as line.

Therefore, this study aims to: (1) compare the performances of the instruments with different NIR detectors for the determination of total soluble solid contents of single grape berry; (2) investigate the influence of the number of standardization samples to model transfer and select optimal number of samples for calibration transfer; and (3) study the feasibility using a modified calibration transfer method (linear interpolation-PDS) between a benchtop Fourier transform (FT) spectral spectrometer and a portable grating scanning spectrometer. 

## 2. Materials and Methods

### 2.1. Intact Berry Samples

In this study, 70 clusters of *Vitis vinifera* L. cv. “Ruby Seedless” (the generation variety of *Vitis vinifera* L. cv. “Emperor” and *Vitis vinifera* L. cv. “pirovan075”) were manually harvested in October 2015 from three different vineyards (32°5′ N, 118°8′ E, NanJing, China). Then, a total of 10 berries were randomly picked from the top, middle and bottom of each cluster, so 700 grape berries of “Ruby Seedless” were employed for the study. To eliminate temperature effects, all samples were allowed to equilibrate at ambient temperature (20 ± 0.5 °C) for 30 min before performing the experiment.

### 2.2. Spectral Collection and Reference Methods of SSC

The intact grape berries were put into sample cell of each instrument one by one. The reflectance spectrum of each sample was obtained with 32 scans. Each sample was scanned three times, and the average spectrum was used for chemometric analysis. The two instruments used for the spectral data collection were:

(i) A benchtop Fourier transform (FT) spectrometer (VECTOR 22/N, Bruker Optics, Germany), equipped with a deuterated triglycine sulfate detector (DTGS) detector covering the spectral range from 12000 to 4000 cm^−1^ (833–2500 nm), and the spectral resolution of this spectrometer is 3.858 cm^−1^.
(ii) A portable grating scanning spectrometer (SupNIR-1500, Focused Photonics Inc., Hangzhou, China) equipped with an InGaAs detector and a 3.4 cm diameter clear aperture, with the spectral range between 1000 to 1800 nm and 1 nm wavelength increments.

The reference value of SSC was measured by a digital hand-held “Pocket” refractometer (PAL-1, ATAGO, Minato-ku, Japan), and was expressed in Brix (%), with accuracy of 0.1% unit. Each sample was determined in triplicate, and the mean of three measurements was utilized for subsequent analysis. The standard error of laboratory (SEL) was calculated as follow:

$$\mathrm{SEL}=\sqrt{\frac{\sum^{\text{​}}{\left(y_{1}-\bar{y}\right)}^{2}+{\left(y_{2}-\bar{y}\right)}^{2}+{\left(y_{3}-\bar{y}\right)}^{2}}{N}}.$$

*y*_1_, *y*_2_*,* and *y*_3_ being the values obtained for a sample and its repetition, and $\bar{y}$ being the mean of three measurements. *N*, the number of samples used to calculate the SEL. 

The reference values were analyzed by IBM SPSS statistic 22 software (IBM Inc., New York, NY, USA). 

### 2.3. PLS, LS-SVM Regression 

The spectra were preprocessed by moving-average smoothing (MS) and standard normal variate (SNV) to reduce the noise and the interferences of scatter and particle size [16]. Then the Kennard-Stone (KS) [17] algorithm was utilized to select calibration set (550 samples) and prediction set (150 samples). The KS algorithm calculates the Euclidean distances of every two spectra and selected two spectra with the furthest distance as starting pair, then calculates the Euclidean distances of the rest spectra with the starting pairs, which made the samples were representative in both sets and could prevent over-fitting or less-fitting to some extent.

In this study, partial least squares (PLS) [18] and the least-squares support vector machine (LS-SVM) [19] regression were conducted to predict the SSC of grape berries. Partial least squares regression is a shared, simple linear method for the investigation of spectral and reference values. Using the cross-validation approach, the optimum PLS model was determined by selecting the first minimum value from the prediction residual error sum of squares (PRESS) curve, and it could be determined whether there was any over-fitting or not. The least-squares support vector machine (LS-SVM) handles both linear and nonlinear relationships between the spectra and chemical components, and radial basis function (RBF) was used, and the parameters gam (γ) and sig^2^ (σ^2^) were selected by leave-one-out-cross validation (LOOCV). 

### 2.4. Passing-Bablok Regression

To verify the differences between prediction values of each instrument and reference values are statistically significant, Passing-Bablok regression [20] was conducted on the prediction set over the common range of 1000–1800 nm, and based on the values of slopes and intercepts, optimal instrument will be chosen as master instrument. The slope and intercept are calculated with their 95% confidence interval. The slope is a measure of the proportional differences, and when the confidence interval for slope contains the value 1, then it is concluded that the hypothesis (slope = 1) is accepted; The intercept is a measure of the systematic differences, and when the confidence interval for intercept contains the value 0, then the hypothesis (intercept = 0) is accepted. In conclusion, H_0_ is accepted when slope = 1 and intercept = 0 are both accepted at a 95% confidence level.

### 2.5. Mean Normalization and Standardization Samples Selection

Mean normalization was performed to reduce the differences between two different instruments before conducting model transfer. Mean normalization is useful for several groups of data at different order of magnitude to be compared by getting all data in approximately the same scaling. 

Before model transfer, the spectra of a few, representative samples should be selected to establish the relationship between master and slave instruments. Feudale [21] pointed out the importance of selecting optimal standardization samples and appropriate quantity of standardization samples in his review. However, few papers had taken the number of standardization samples into consideration when applying calibration transfer. In this study, the KS algorithm was also utilized to select standardization samples. In order to investigate the influence of the number of standardization samples to calibration transfer, the different quantity of standardization samples were selected from calibration set and the optimal quantity of standardization samples were determined by the lowest root mean squares error of prediction set. Besides, whether or not the standardization samples should be taken into calibration set was also discussed here by recalculating the model performances without standardization samples in calibration set.

### 2.6. Linear Interpolation-PDS for Model Transfer 

The theory of piecewise direct standardization (PDS) is as follows:

The response matrix dimensioned samples by wavelengths of standard set are chosen at wavelength index *i* on the master instrument, called *R_m,i_*.

The response matrix dimensioned samples by wavelengths of standard set at wavelength index *i* − *j* to *i* + *k* on slave instrument are chosen, called *R_s,i_*.

(1) $$R_{m,i}=R_{s,i}\times b_{i}$$

The regression vector *b_i_* can be calculated via PLS. Then the transformation matrix F can be calculated by setting the off-diagonal elements to zero. That is:

(2) $$F=\mathrm{diag}\left(\;b_{1}^{T},b_{2}^{T},\cdots ,b_{i}^{T},\cdots ,b_{n}^{T}\;\right).$$

Here, *n* is the number of wavelengths.

The PDS performed well in the application of model transfer between different instruments of the same type or between instruments of different types with same number of wavelengths. However, when facing different instruments of different types as well as with different number of wavelengths, this method may be unsuitable. Traditional method to solve this problem is to: reserve the common wavelengths of both instruments which depressed the wavelengths and resulted in information missing from spectra; or using DS to take place of PDS by standardizing a range of frequencies to the response of master instrument at the entire spectrum which would result in enormous computation. Thus, we proposed a modified PDS to solve this problem, and this linear interpolation-PDS can solve wavelength correction problems. We added a process of wavelength correct combined photometric correction before PDS. In the approximately same spectral range of master instrument and slave instrument, the spectra matrix of slave instrument is converted to match that of master instrument by computing the spectral values at nearby wavelengths for each sample via first linear equation:

(3) $$Slope_{c}=\frac{y_{s,j\;}-y_{s,i}}{j-i}$$

(4) $$b_{c}=y_{s,j}-Slope_{c}$$

(5) $$y_{s,c}=Slope_{c}\times c+b_{c}.$$

Here, *c* is a wavelength of master instrument; *i* and *j* are the nearest wavelength to wavelength *c* on the slave instrument; *y_s,j_* is the response values of each sample at wavelength *j*, and *y_s,i_* is response values of each sample at wavelength *i*; the response values of slave instrument at wavelength *c* is generated, called *y_s,c_*.

### 2.7. The Model Evaluation

The performances of different models mentioned above were evaluated by the determination coefficient of calibration (R_c_^2^), determination coefficient of prediction (R_p_^2^), the root mean squares of calibration (RMSEC), prediction (RMSEP), and the ratio of standard deviation to standard error of prediction (RPD). For a promising model, higher values of R_c_^2^, R_p_^2^ and RPD along with lower values of RMSEC and RMSEP should be achieved simultaneously. In addition, over-fitting should be avoided during the calibration. Thus, the RMSEC must be lower than RMSEP, yet the difference between the two values cannot be significant. 

All data processing was conducted with MATLAB 2010b (The Mathworks, Natick, MA, USA).

## 3. Results and Discussion

### 3.1. Statistics of SSC

**Figure 1:**
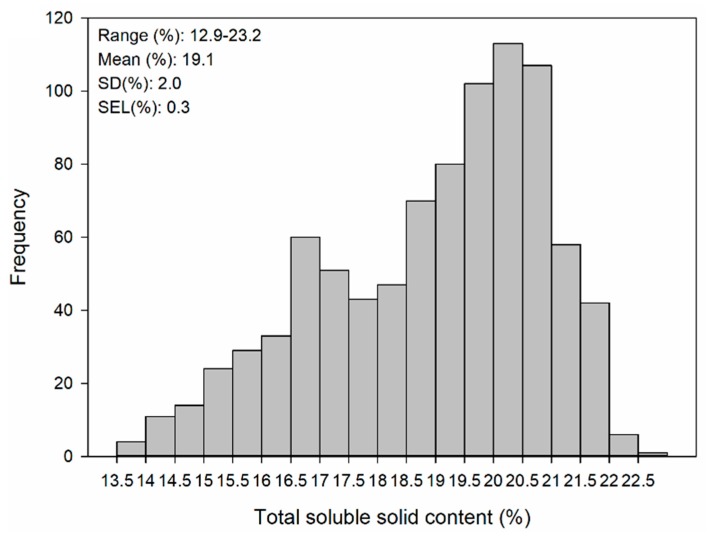
Distributions of total soluble solid contents in single grape berries for Ruby Seedless with range, mean value, and standard deviation (SD).

The distribution of total soluble solid contents of Ruby Seedless is shown in Figure 1. The statistical values were between 12.9% and 23.2%, and the average value was 19.1%. The standard deviation of references (SD) was 2.0%, and the SEL was 0.3%. The one-sample Kolmogorov-Smirnov test showed that the Skewness values and Kurtosis values of the SSC was lower than 1. The berry samples covered a broad range of concentrations and the results of one-sample Kolmogorov-Smirnov test indicated that the statistical values of SSC were roughly in a normal distribution. Also, the wide variability of the data is helpful for developing a stable and robust calibration model. The results obtained here were in accordance with those of Parpinello [6] (SSC ranges from 11.8–22.6%) and Urraca [22] (SSC ranges from 10.3–21.6%). 

### 3.2. Spectra Preprocessing

**Figure 2:**
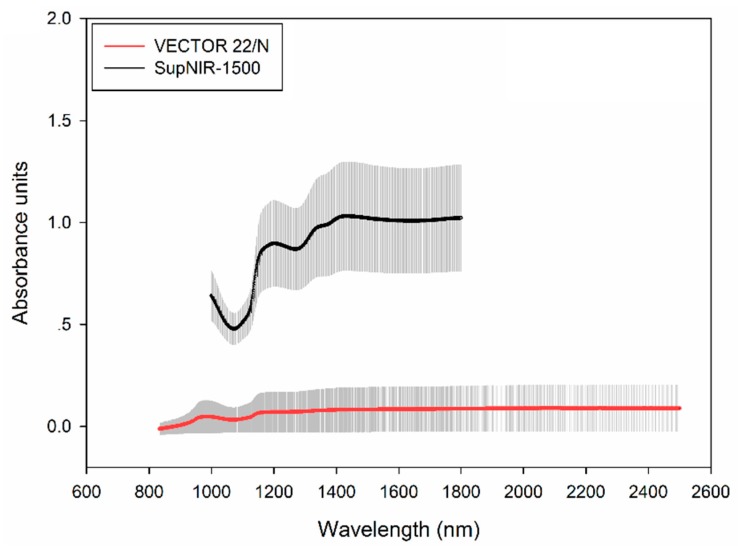
The raw average spectra with standard deviation of Ruby Seedless.

The raw spectra (average spectra with standard deviation) of grape berries are shown in Figure 2. As can be seen, spectral shapes over the same wavelength range (1000–1800 nm) for two instruments were similar, and prominent absorptions peaks at around 1050 nm, 1185 nm, 1450 nm, and 1770 nm are probably assigned to 3 × O-H stretching vibration of water and sugars, 2 × C-H stretching vibration combined with 2 × C-H deformation vibration, C-H overtone in sugars and organic acids, C-O stretching vibration or overtone band in sugars or organic acids, 2 × C-H stretching vibration of sugars, acids, and water, respectively [23].

The performances of pretreatments were evaluated by cross-validation using PLS regression. Considering the optimized RMSECV and highest RPD, it was found after several trials that the best pretreatments were: standard normal variate (SNV) and 18 points of moving-average smoothing (MS) for spectra acquired from VECTOR 22/N; SNV and three points of MS for spectra acquired from SupNIR-1500. All regression models were developed after conducting pretreatments of moving-average smoothing (MS) and the standard normal variate (SNV) to reduce noise and potential interferences of scatter and particle size. 

### 3.3. SSC Prediction of Grape Berries

Statistics of PLS and LS-SVM regressions after pretreatments are shown in Table 1. The results obtained showed that the LS-SVM models with R_p_^2^ of 0.889–0.918 and RPD of 2.191–2.536 performed better than the PLS models with 0.874–0.907 and 2.062–2.396 for R_p_^2^ and RPD, respectively, and the RMSEC as well as RMSEP greatly decreasing. All of the RMSEC and RMSEP values of regression models were slightly higher than SEL, but did not show large differences, indicated that the error of NIR prediction was acceptable in this work [24]. Furthermore, the RPD values of LS-SVM model were higher which indicated that the regression was more reliable. And these results were in accordance with the results predicted by Paroinello [6] who determined the SSC of table grape by a Fourier Transform instrument with the lowest RMSECV of 1.35% and the highest R_p_^2^ of 0.93.

PLS is one of the commonly used chemometric methods in the prediction of quality parameters including ergosterol [25], total phenolic compounds [26], titratable acidity [27] in grapes, and all the established PLS models presented fairly good results in these studies. Fernández-Novales [28] applied PLS for the prediction of reducing sugar contents at different stages of grape ripening, winemaking, and aging of red and white wines with an excellent R_p_^2^ of 0.988. Chauchard [29] found that LS-SVM performed better than PLS for the prediction of acidity of grapes. LS-SVM also performed better than PLS regression in the prediction of chemical parameters of acerola [30]. The LS-SVM regression took all the linear and nonlinear relationships into consideration while PLS could only solve liner problems, however, there does not seem to be convincing evidence to prove that the non-liner method offers more advantages than the liner method [23]. Here, considering the LS-SVM models performed better than PLS in this work, LS-SVM models were used for the follow-up discussion.

The models with data in the 830–2500 nm region obtained by VECTOR 22/N were found to be the best with R_c_^2^ of 0.985, R_p_^2^ of 0.918, and highest RPD of 2.536 which can be considered as excellent. The spectral range of 830–2500 nm performed better than the range of 1000–1800 nm on VECTOR 22/N with higher R_p_^2^ and RPD, along with lower RMSEP, which probably due to the wider NIR range contains more information related to the soluble solid contents of grapes. Fragoso [31] compared the full-range spectra (979–2989 nm), fingerprint spectra (979–1477 nm), main phenolic region (1133–1457 nm), and selected region (1168–1457) for the prediction of phenolic compounds in grapes, found that in most cases, full-range spectra acquired the best prediction results.

However, when comparing the performances of two instruments in the same wavelength range (1000–1800 nm), SupNIR-1500 did better in the evaluation of SSC. The big differences between the models can probably be explained by the differences between the devices, due to the different instrumental parameters such as resolution, type of monochromator and detector. Even though the preprocessing methods decreased the noise, some noise and redundancy information still exists. It is noteworthy that all of the RPD values of LS-SVM models were higher than 2.0, confirming that the LS-SVM models were very efficient in generating the predictions for the SSC of grape.

The results of Passing-Bablok regression for LS-SVM are shown in Table 2. The null hypothesis (H_0_) accepted means that based on a 95% confidence level, the slope between the two compared groups is not significantly different from 1 and that the intercept between the two groups is not different from 0. The results indicated that there exists difference between the predicted values of VECTOR 22/N and reference values for Ruby Seedless, and the prediction by SupNIR-1500 is more reliable. Thus, SupNIR-1500 was regarded as master instrument in the calibration transfer analysis. And the calibration transfer were conducted on the LS-SVM models.

### 3.4. Calibration Transfer

**Figure 3:**
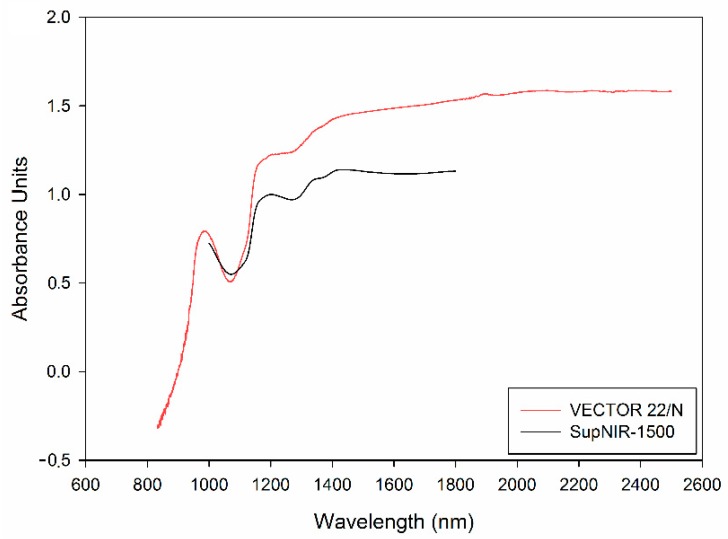
The average spectra conducted mean normalization of Ruby Seedless.

The average mean-normalized spectra of Rube Seedless are shown in Figure 3. The main spectral features were retained and the differences between two instruments were more visible. In the meanwhile, the data of two instruments in the same spectral range of 1000–1800 were in the same scaling, which is helpful for calibration transfer.

**Figure 4:**
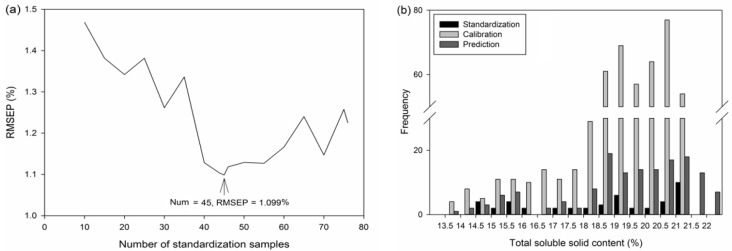
The plot of the number of standardization verses the root mean squares error of same prediction set based on least-squares support vector machine regression (**a**) and the distributions of total soluble solid contents in the optimal linear interpolation-PDS model (**b**).

Figure 4a shows the relationship between the number of standardization samples and RMSEP based on LS-SVM. It should be noticed that the standardization samples were included in calibration set (meant that the calibration set was fixed), and the prediction set was fixed. The lowest RMSEP of 1.099% was found when 45 samples were chosen for standardization, and when the number of standardization samples over 76, the model transfer could be regarded as unreasonable with R_p_^2^ lower than 0. It was difficult to explain the reason why choosing 45 samples is sufficient. Chen [32] discussed the effects of calibration sample numbers on NIR model of tea, he found that when 85 samples were calibrated the model was best, and explained it possibly due to the redundant samples gave redundant background information which was unnecessary for model establishment. So we assumed here that when more than 45 samples were selected, the redundant information they provided may affect transfer performance. And this result provided the evidence that the better calibration transfer results could not be achieved by simply using more standardization samples. The distributions of total soluble solid contents in the standardization, calibration, and prediction sets when using 45 standardization samples are shown in Figure 4b. The concentration distributions looked similar in three sets, and this result in turn proved that KS selection ran well in this study.

The transfer performances were re-evaluated when standardization samples were removed from calibration set, and the results are shown in Table 3. The RMSEP showed similar trend with Figure 4a, and it was interesting that the situation when 45 samples were selected for standardization set turned out to be the optimal transfer result, which in accordance with the results of Figure 4a. However, the lowest RMSEP of 1.214% in Table 3 was higher than that of 1.099% in Figure 4a. To the best of our knowledge, there are limit papers discussed whether or not the standardization samples should be calculated in master model. In most cases, the standardization samples were chosen from prediction set, and then they were re-predicted by master model [33]. Our study proved that re-calculated the standardization into mater model could improve the performances of calibration transfer to some extent.

Here, linear interpolation-PDS was compared with linear interpolation spectra without PDS (using linear interpolation to wavelengths of slave spectra matrix, but did not using PDS to eliminate the instrument differences) and the common-wavelengths-reserved-PDS, and prediction using standard spectra was based on LS-SVM. The response of VECTOR 22/N was converted to fit the response of SupNIR-1500 at each wavelength before linear interpolation-PDS and finally the data matrixes of each instrument contain a total of 800 wavelengths; a total of 230 common wavelengths for both instruments were selected before common-wavelengths-reserved-PDS, and all of these wavelengths were taken into the consideration of PDS. After several trails, the optimal half-width of the window for PDS was selected by lowest RMSEP using a total of 45 standardization samples. The results are shown in Table 4. Although the results of linear interpolation-PDS were not better than that of master spectra, this method produced better results than common-wavelengths-reserved-PDS, with considerably higher R_p_^2^ of 0.857 and RPD of 1.895, along with lower RMSEP of 1.099%. 

**Figure 5:**
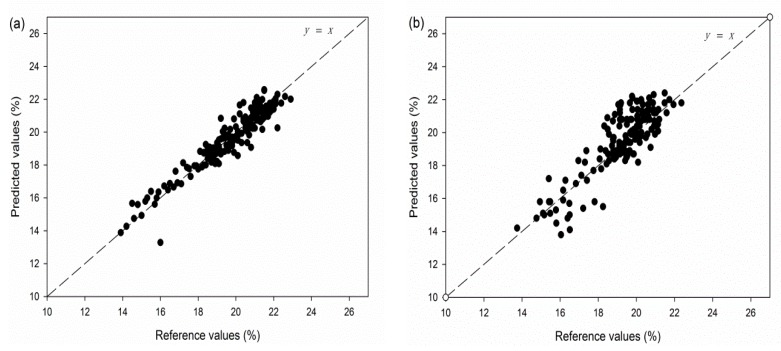

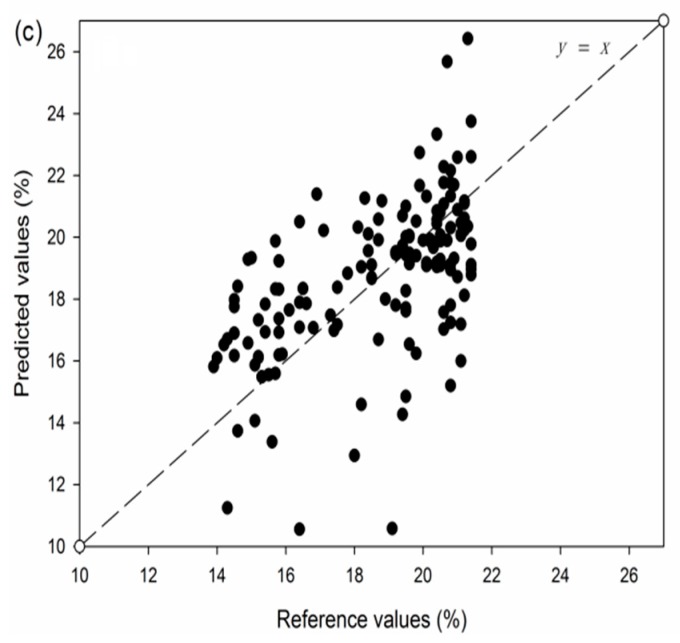
The plots of the predicted values verses the reference values of prediction sets based on least-squares support vector machine regression ((**a**) master instrument; (**b**) linear interpolation-PDS; (**c**) common-wavelengths-reserved-PDS).

**Figure 6:**
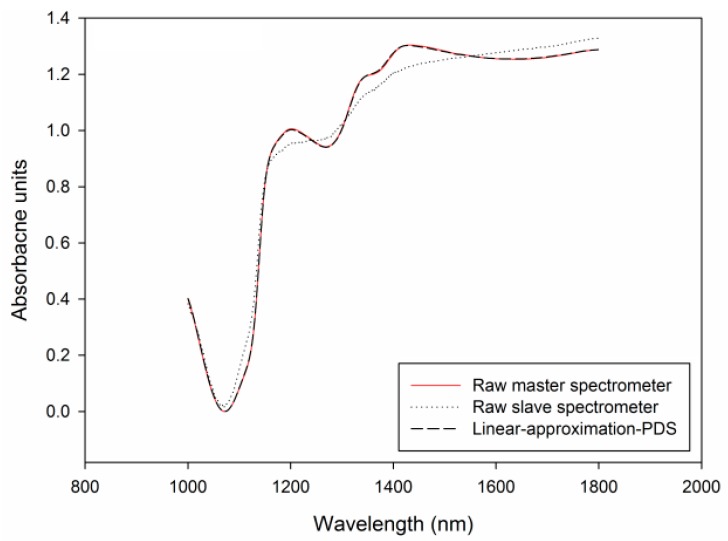
The average spectra of prediction sets for Ruby Seedless in the range of 1000–1800 nm.

The plots of the predicted values verses the reference values of different models are presented in Figure 5. In these figures, the straight lines which pass through the origin were linear-curve fitting results between the references and predicted values which represent the relationship between the two. And when the slope is near to 1, means that predicted values were close to reference values. From the plots we found that the slope of linear interpolation-PDS is more similar with that of master set, it in turn indicted that linear interpolation-PDS performed better than common-wavelengths-reserved-PDS. Figure 6 shows the average spectra of master instrument, original slave instrument and transferred slave instrument of same prediction set. It could be seen that transferred spectra of slave instrument looked more similar to the original spectra of master instrument.

The linear interpolation-PDS combines advantages of PDS and Shenk’s Algorithm which takes wavelength correction into consideration. Moreover, the linear interpolation-PDS solves the enormous computation of multiple regression at each wavelength in PDS algorithm and solves transfer problems between different NIR spectrometers with different wavelength intervals; and it takes the relationship between wavelength and photometric into consideration while Shenk’s Algorithm does not. Pérezmarín [34] pointed out the importance of wavelength correction when using Shenk’s Algorithm to transfer the ingredient composition calibration model from a Foss NIRSystem 6500 SY-II (400–2500 nm) to a Foss NIRSystems 5000 (1100–2500nm). Sulub [35] and Peng [36] found that PDS performed well in the calibration transfer between different NIR spectrometers with the same wavelength intervals. PDS performed better than direct standardization (DS), orthogonal signal correction (OSC), reverse standardization (RS), piecewise reverse standardization (PRS), slope and bias correction (SBC) in the prediction of quality parameters for gasoline between a FT-IR PerkinElmer Spectrum GX spectrometers and an ABB Bomen FT-NIR MB160D spectrometer in Pereira’s [37] work. Liang [12] proposed a Rank-KS algorithm combined PDS to acquire a better calibration transfer method. By combining the KS-PDS with our linear interpolation hypothesis for variables selection and conversion, a better transfer results were obtained in the prediction of SSC for grape berries between a benchtop Fourier transform (FT) spectrometer and a portable grating scanning spectrometer.

## 4. Conclusions

In this study, total soluble solid contents of Ruby Seedless were predicted by two totally different instruments, considering the alignment of wavelengths and selection of standardization samples, a modified PDS transfer method is generated to transfer the calibration between a benchtop Fourier transform (FT) spectrometer and a portable grating scanning spectrometer. And this linear interpolation-PDS can solve the difficulty caused by resolution difference and performed better than traditional wavelengths-reserved method. And it was proved that re-calculating the standardization samples into master model could improve the performance of calibration transfer in this study.

## Figures and Tables

**Table 1:** Statistics of partial least squares (PLS) and least-squares support vector machine (LS-SVM) regressions for total soluble solid contents of Ruby Seedless.

Methods	Devices	R_c_^2^	RMSEC (%)	R_p_^2^	RMSEP (%)	RPD
PLS	VECTOR 22/N	0.963	0.515	0.888	0.889	2.168
	VECTOR 22/N-P	0.928	0.714	0.874	0.935	2.062
	SupNIR-1500	0.941	0.645	0.907	0.811	2.396
LS-SVM	VECTOR 22/N	0.985	0.340	0.918	0.758	2.536
	VECTOR 22/N-P	0.959	0.557	0.889	0.878	2.191
	SupNIR-1500	0.969	0.477	0.910	0.801	2.420

R_c_^2^: Determination coefficient of calibration; R_p_^2^: Determination coefficient of prediction; RMSEC: Root mean squares error of calibration; RMSEP: Root mean squares error of prediction; RPD: Ratio of standard deviation to standard error of prediction; VECTOR 22/N-P: The spectra in the range of 1000–1800 nm on VECTOR 22/N.

**Table 2:** Passing-Bablok regression results for least-squares support vector machine of total soluble solid contents prediction at 95% confidence level.

Cultivar	Parameters	SupNIR-1500 vs. Reference	VECTOR 22/N vs. Reference
Ruby Seedless	Intercept	−1.7832 to 0.1420	−5.5442 to −1.5561
	Slope	0.99953 to 1.0915	−1.0790 to 1.2781
	H_0_	Accepted	Rejected

H_0_: The null hypothesis.

**Table 3:** Re-calibration of total soluble solid content for Ruby Seedless when standardization samples were removed from calibration set and model transfer performances using linear interpolation-PDS LS-SVM.

Num	R_c_^2^	RMSEC (%)	R_p_^2^	RMSEP (%)	RPD
10	0.952	0.546	0.716	1.478	1.408
15	0.959	0.502	0.745	1.514	1.375
20	0.955	0.525	0.791	1.390	1.497
25	0.964	0.472	0.773	1.421	1.465
30	0.954	0.525	0.802	1.339	1.554
35	0.957	0.508	0.765	1.446	1.439
40	0.951	0.538	0.841	1.231	1.691
42	0.954	0.517	0.841	1.231	1.690
43	0.963	0.467	0.841	1.217	1.710
44	0.956	0.510	0.835	1.259	1.654
45	0.957	0.506	0.856	1.210	1.714
46	0.956	0.508	0.849	1.242	1.676
47	0.955	0.514	0.849	1.254	1.660
50	0.963	0.471	0.836	1.241	1.677
55	0.954	0.521	0.830	1.258	1.655
60	0.961	0.484	0.828	1.300	1.601
65	0.961	0.480	0.797	1.387	1.501
70	0.954	0.521	0.815	1.290	1.614
75	0.956	0.511	0.806	1.300	1.603

Num: Number of standardization samples; R_c_^2^: Determination coefficient of calibration; R_p_^2^: Determination coefficient of prediction; RMSEC: Root mean squares error of calibration; RMSEP: Root mean squares error of prediction; RPD: Ratio of standard deviation to standard error of prediction; PDS: Piecewise direct standardization; LS-SVM: Last-squares support vector machine.

**Table 4:** Performances of calibration transfer methods for the total soluble solid contents of Ruby Seedless based on least-squares support vector machine (LS-SVM).

Methods	R_p_^2^	RMSEP (%)	RPD
Origial	0.125	28.487	0.072
Common-wavelengths-reserved-PDS	0.471	3.489	0.676
Linear interpolation-PDS	0.857	1.099	1.895

R_p_^2^: Determination coefficient of prediction; RMSEP: Root mean squares error of prediction; RPD: Ratio of standard deviation to standard error of prediction; PDS: Piecewise direct standardization.
